# Integrated genomics and morphological approach reveals interspecific gene flow cases and decodes the origin of selected feathergrasses (Poaceae, *Stipa*)

**DOI:** 10.1038/s41598-025-08934-y

**Published:** 2025-10-01

**Authors:** Patar Sinaga, Ewelina Klichowska, Serik Kubentayev, Marcin Nobis

**Affiliations:** 1 Institute of Botany, Faculty of Biology, Jagiellonian University, Gronostajowa 3, 30-387 Kraków, Poland; 2 Doctoral School of Exact and Natural Sciences, Jagiellonian University, Kraków, Poland; 3 Astana Botanical Garden, Orynbor 16, Astana, 010000 Kazakhstan

**Keywords:** Integrative taxonomy, Molecular evidence, Morphology, Natural hybridisation, *Stipa*, Evolution, Plant sciences

## Abstract

Central Asia is a diversity hotspot of arid-adapted grasses from the genus *Stipa*, with approximately 100 taxa found in the region. Recent studies in the steppe areas of Kazakhstan revealed specimens displaying intermediate morphology, distinguishing them from other taxa that grow sympatrically. Using integrative taxonomy, we investigated whether these individuals resulted from natural speciation or hybridisation, and if so, we would like to know which species were involved in this process feathergrasses. Research conducted in steppes of central Kazakhstan (Kyzylorda region), revealed the existence of individuals morphologically intermediate between *S. arabica* and *S. richteriana*, suggesting that these are probably of hybrid origin. Morphology and SNP markers validated the specimens as F1 hybrid between the aforementioned species by cladding separately based on neighbor-joining phylogenetic tree. Moreover, genetic structure displayed a separate cluster and showed almost equal genetic admixture between *S. arabica* and *S. richteriana*. Additionally, fastStructure analysis detected two geographically separated cryptic genotypes within *S. richteriana* population and their involvement in the hybridisation resulted in occurrence of *S*. × *heptapotamica*, *S.* × *czerepanovii* and *S*. × *korshinskyi* which recently were suggested as hybrids. Based on these evidences, we described a new nothospecies *S*. × *kyzylordensis*, as F1 hybrid. Furthermore, morphologically, the nothospecies delimited with other hybrids in Kazakh steppe area, marking the first report of hybridisation between *S. arabica* and *S. richteriana*, along with molecular evidence for the origin of further species supposed to be hybrids. This finding is crucial to understanding species diversity and hybridisation process in morphologically and genetically distant *Stipa* species.

**Supplementary Information:**

The online version contains supplementary material available at 10.1038/s41598-025-08934-y.

## Introduction

One of the largest and most ecologically and economically significant plant groups is the grasses family (Poaceae), with 11,783 species in 789 recognised genera^1^. Based on most recent phylogenomic studies, grasses are believed to have originated in the Early–Late Cretaceous, ca. 100 Mya^2,3^. During Cenozoic, Poaceae has diversified into the fifth most species-rich angiosperm family, showing tremendous evolutionary success^4,5^. According to Bouchonak-Khelladi et al.^6^, their evolutionary history started in the understory of tropical forests or along forest borders, where they may move from completely shaded to open habitats. Diversification of numerous species-rich grass lineages was driven by independent shifts to open environments that began in the Paleocene^7^, while reversals back into forest understories were less common. Grasses have an important role in ecosystems all across the world, as evidenced by its widespread and high species variety. Representatives of the family span up to 40% of the total area of the Earth and are found in a variety of habitats on all major continents, including croplands, tropical savannas, and temperate grasslands^8^.

In the mechanism of Poaceae evolution, hybridisation has played a significant role by driving genetic diversity and shaping the evolutionary history of species^9^. It is well-known to be especially frequent in connection with polyploidy and within polyploid complexes, as documented in many grass groups^10,11^. In natural populations, hybridisation serves as an evolutionary catalyst by facilitating the removal of reproductive barriers^12^. Up to 25% of plant species are thought to naturally hybridise, which is a typical occurrence in the kingdom of plants^13^. Hybridisation and introgression increase the genetic diversity of plant populations by transferring adaptive potential^14^. Furthermore, it has been demonstrated that hybridisation is an important factor in speciation, promoting the emergence of new species with greater genetic and adaptive diversity^15^. However, hybridisation can also lead to reduced fitness and genetic swamping^16,17^. Through reinforcement, hybridisation can increase reproductive isolation when maladapted hybrids are targeted by natural selection^18^.

Distant hybridisation, involving crosses between distantly related plant species, enhances genetic diversity by fostering novel genotypic and phenotypic traits through genome recombination, contributing to adaptive radiations and new evolutionary pathways^19^. Distant hybridisation crosses species boundaries by allowing genome transfer and causes essential changes in the genotype and phenotype of progeny^20^. According to Chen et al.^20^, true hybrids frequently display a combination of parental characteristics as well as distinctive ones, proving their hybrid origin.

*Stipa* L., a genus within the Poaceae family, exemplifies the significance of hybridisation in plant evolution. In general, *Stipa* is distributed across Europe, North Africa, and Asia showing its exceptional adaptation to dry temperate climates. In Central Asia alone, *Stipa* consisted over 76 taxa (species and subspecies), classified as endemic, subendemic or rather wide-distributed^21^. The genus occupies a range of habitats, from open grasslands to rocky and sandy deserts^21,22^. Around 30% of the 150 identified *Stipa* species are hybrids^21^. *Stipa* hybrids are perennial species with the ability to produce viable pollen and can reproduce vegetatively by clonal reproduction, which allows for backcrossing with parental taxa, thus increasing the potential for introgressive hybridisation and further contributing to the genetic complexity of this genus^22–26^.

During fieldwork in the lowlands of central Kazakhstan, we encountered specimens displaying morphological characters that did not correspond to any of the known species in the region, which includes *Stipa arabica*, *S. hohenackeriana*, *S. sareptana*, *S. richteriana*, and *S. caucasica* var. *fanica* growing there. These specimens exhibited specific morphological characters, making it challenging to classify them within co-occurring populations of *Stipa* taxa. In this study, we aim to answer the question of whether these specimens represent a new species, a new hybrid of the co-occurring species or rather, these phenotypes represent only morphological variation (plasticity) of one of the co-occurring species. Whereas, if they are hybrids, how do they differ from other similar species of hybrid origin known from other regions of Kazakhstan? To meet these objectives, we conduct an integrative taxonomy approach by combining an extensive examination of morphological characters (quantitative and qualitative) and molecular analysis based on DArTseq-based genome-wide sequencing.

## Material and methods

### Ethics statement

Plant material collection complied with institutional, national and international regulations. The collecting location reported in this work is not protected area by any law. Plant samples were collected exclusively on public land. In addition, the plant species collected here are neither endangered nor protected. No specific permits were required for this study.

### Field study

A field study was conducted in Kazakhstan’s steppe region in 2019 and 2022. As a separate, we treated localities that were approximately 5 km or more apart from each other. We collected *Stipa* in Kyzylorda and Ulytau regions (central Kazakhstan), where we found *S. arabica* Trin. & Rupr. *S. caucasica* var. *fanica* M. Nobis, P. D. Gudkova & A. Nowak, *S. hohenackeriana* Trin. & Rupr., *S. sareptana* A. K. Becker, *S. richteriana* Kar. & Kir., and putative new taxon. This study area is located in the zone of northern Eurasian deserts belonging to continental arid regions with temperate (subboreal) climate. The landscape is represented by extensive clay plains occupied by desert vegetation complexes dominated by typical xerophytic species*: Artemisia terrae-albae* Krasch., *A. lercheana* Kar. & Kir., *Oreosalsola arbusculiformis* (Drobow) Sennikov, *Stipa richteriana*, *S. kirghisorum* P.A. Smirn. and *Poa bulbosa* L. Along with them, biyurgun-tasbiyurgun communities formed by *Nanophyton erinaceum* (Pall.) Bunge and *Anabasis salsa* (Ledeb.) Benth. ex Volkens, growing on brown and associated soils, typical for this natural zone, are widespread. Among shrubs *Calligonum aphyllum* (Pall.) Gürke and *Haloxylon ammodendron* (C.A.Mey.) Bunge ex Fenzl^27–29^.

Fresh plant samples (leaves) were collected to silica gel, whereas the specimens are preserved at the KRA herbarium (Herbarium of the Jagiellonian University in Kraków). All samples were subsequently used in our morphological and molecular analyses. The location, coordinates, and altitude of the sampling sites were described and recorded using GPS coordinates.

### Plant material

Morphological and molecular studies were based on 91 specimens, of which 13 samples belong to *S. arabica*, 12 to *S. caucasica* var. *fanica*, 12 to *S. hohenackeriana*, 20 to *S. sareptana*, 24 to *S. richteriana*, and 10 to putative new taxon. Nomenclature of species follows Nobis et al.^21^. Despite having the same number of specimens for both analyses, there are slight differences in the compositions of specimens per species. This is due to not all specimens for morphological measurements had their DNA sequenced, so additional samples for molecular analyses were included for molecular analyses (Table S1). In addition to the six aforementioned taxa, morphological identification was also conducted on four *Stipa* species of hybrid origin: 18 samples of *S*. × *heptapotamica* Golosk. (*S. lessingiana* Trin. & Rupr. × *S. richteriana*), 13 of *S*. × *czerepanovii* Kotukhov (*S. orientalis* Trin. × *S. richteriana*), 4 of *S*. *korshinskyi* Roshev. (possibly a hybrid between *S. sareptana* × *S. richteriana* subsp. *richteriana* as suggested by Nobis et al.^21^) and 1 sample of *S*. × *pseudocapillata* Roshev. (*S. capillata* L. × *S. lessingiana*). They have all been described based solely on morphology (Nobis et al.^21^), and the last two of them still require molecular confirmation. The four above-mentioned hybrids were included in the comparative analysis since the newly recognised morphotype displays morphological similarity to them. Moreover, all hybrids and their potential parental species are common in the study area and often co-occur with the putative new taxon. Besides the specimens collected from the population occurring in central Kazakhstan, for comparative purposes, we included also specimens representing particular mentioned above taxa from other areas of Kazakhstan (outside of the Kyzylorda region; Supplementary Table S1). Whereas for widely distributed species (e.g., *S. capillata*), sampling was supplemented with herbarium specimens collected from other areas of their geographical range.

### Micromorphology

Micromorphological structures of the lemma, callus, and leaf surfaces were studied in all of the examined species, but with a special focus on the putative new taxon. Samples were coated with gold using a JFC-1100E Ion sputter and photographed with a Hitachi S-4700 Cold Cathode Field Scanning Electron Microscope with a NORAN NSS EDS system at various magnifications.

### Morphological data analyses

For each fully developed sample, 51 morphological traits were assessed, comprising 44 quantitative and seven qualitative characteristics (Table 1). Some of the characters were measured on the vegetative shoots of each individual (Table 1) while the rest were measured on the generative shoots. The list of species and specimens used in this study is presented in Table S1. Each sample was treated as an Operational Taxonomic Unit (OTU), following the approach commonly used in numerical taxonomy^30^. Quantitative characters were visualised using boxplots computed in R^31^. To ascertain the univariate normality distribution, the Shapiro–Wilk test was conducted using the R package MVN^32^ after measuring all samples. Subsequently, descriptive statistics of characters for all recognised groups were calculated. To reveal significant differences between means of characters across all examined groups (after using Levene’s test to assess the equality of variances), depending on the accepted assumption for normal distribution and equality of variances, one-way ANOVA or non-parametric Kruskal–Wallis test were calculated using PAST 4.03^33^. Principal Component Analyses (PCA) were carried out in STATISTICA 13.3^34^ and R based on the quantitative characters with factor loadings ≥ 0.6. Clustering analysis in the form of UPGMA dendrogram was carried out in PAST 4.03 based on Gower’s similarity index.

**Table 1:** Quantitative and qualitative characters for morphological examination.

Code	Morphological characters
Quantitative characters in millimeters (except for characters with *)	
LCL	Lower column length
UCL	Upper column length
CW	Column width
CH	Length of hairs on column
SL	Seta (upper segment of the awn) length
Awn	Awn length
SH	Length of hairs on seta
Ratio S/C*	Ratio of seta length to column length
Ratio SH/CH*	Ratio of length: seta hairs to column hairs
AL	Floret (= anthecium) length
AW	Floret (= anthecium) width
LHD	Length of dorsal hairs on lemma
LHV	Length of ventral hairs on lemma
DDL	Distance from the end of the dorsal line of hairs to the top of the lemma
DVL	Distance from the end of the ventral line of hairs to the top of the lemma
HC	Corolla hairs length
CalL	Callus length
CalW	Callus width
CvH	Length of ventral hairs on callus
CdH	Length of dorsal hairs on callus
CRL	Callus foot ring length
CRW	Callus foot ring width
PanicleL	Length of panicle
PanicleW	Width of panicle
UG	Upper glume length at the end of panicle
LG	Lower glume length at the end of panicle
Ratio L1/U1*	Ratio of length: lower to upper glume
Culm	Culm length
Node dist	Distance of the lowest node to the next node
Node*	Number of nodes
NodeHU	Length of hairs on the upper side of the node
NodeHL	Length of hairs on the lower side of the node
BladeL (Cauline)	Length of blade of cauline leaf
BladeW	Width of blade of cauline leaf
SheathW	Upper culm’s sheath width
LL	Vegetative leaves length
LW	Vegetative leaves width (diameter)
LWExp	Vegetative leaves width (expanded)
LAH	Length of hairs on adaxial surface of vegetative leaf
LBH	Length of hairs on abaxial surface of vegetative leaf
LigL1	Length of ligule of inner vegetative shoot
LigH1	Length of hairs on ligule of inner vegetative shoot
LigL2	Length of ligule of outer vegetative shoot
LigH2	Length of hairs on ligule of outer vegetative shoot
Qualitative characters	
Stype	Upper segment of awn (seta) (flexuous = 1, falcate = 2, straight = 3)
CRtype	Callus foot ring (concave = 1, cuneate = 2, pyriform = 3)
GluHU	Upper glume (glabrous = 1, scabrous = 2)
GluHL	Lower glume (glabrous = 1, scabrous = 2)
Tip	Glumes tip (acute = 1, acuminate = 2)
USh	Upper culm sheath (glabrous = 1, scabrous = 2)
MSh	Middle culm sheath (glabrous = 1, scabrous = 2)

### Library preparation, DNA extraction and DArT sequencing

Whole genomic DNA was isolated using Genomic Mini AX Plant Kit (A&A Biotechnology, Poland). NanoDrop One (Thermo Scientific, USA) was used to perform the quantification check. Following the DArTseq methodology, each sample was diluted to a concentration of 50–100 ng/uL. The purified DNA (1–2 μg for each sample) was shipped to Diversity Arrays Technology Pty ltd (Canberra, Australia) for sequencing and marker identification.

DArTseq combines DArT’s complexity reduction techniques with next-generation sequencing technologies. To optimise the complexity reduction strategy for each organism and application, the technology is adjusted accordingly. After testing several enzyme combinations for *Stipa*, Diversity Arrays Technology Pty Ltd. selected the PstI-MseI approach. The reverse adapter included a flowcell attachment region and a MseI-compatible overhang sequence. Only the “mixed fragments” (PstI-MseI) were efficiently amplified through PCR, with an initial denaturation step at 94 °C for 1 min, followed by 30 cycles with the following temperature profile: denaturation at 94 °C for 20 s, annealing at 58 °C for 30 s, and extension at 72 °C for 45 s, finishing with a final extension at 72 °C for 7 min. Following PCR, equimolar amounts of amplification products from each sample in the 96-well microtiter plate were pooled and subjected to c-Bot (Illumina, USA) bridge PCR, then sequenced using Hiseq2500 (Illumina, USA) with 77 single-read cycles.

The sequences from each lane were analysed using proprietary DArT analytical pipelines. Initially, fastq files were processed in the primary pipeline to filter out low-quality sequences, applying stricter selection criteria to the barcode region than to the rest of the sequence. This ensured reliable “barcode split” assignment of sequences to individual samples. Around 2.5 million sequences were identified and used for marker calling per barcode/sample.

### SNPs data filtering and analyses

We filtered initial set of Single Nucleotide Polymorphisms (SNP) markers using the “dartR” package^35^ in RStudio. We provided two analyses with various set of examined species and specimens. In the first one, we examined samples from a population occurring in central Kazakhstan that includes specimens representing *S. arabica-hohenackeriana agg.*, *S. richteriana*, *S. sareptana*, *S. caucasica* var. *fanica* and the new putative taxon. The filtering steps of data were as follows: (1) omitting all monomorphic loci; (2) calculating call rates for each locus (threshold 80%); (3) excluding loci with reproducibility < 1; (4) excluding secondary forms; and (5) filtering loci based on Minor Allele Frequency (threshold 5%). In total, 3,444 SNP loci were generated from 91 samples (Supplementary Table S1). The resulting genlight object was used for generating neighbor-joining phylogenetic tree in package “poppr”^36^ and visualized in Interactive Tree of Life (iTOL) v6^37^. Genetic admixture quantification was implemented in fastStructure^38^. This analysis was utilized to visualize genetic admixture among the five taxa for K values from 1 to 10 in our dataset (*S. arabica-hohenackeriana agg.*, *S. richteriana*, *S. sareptana*, *S. caucasica* var. *fanica* and the new taxon). Additionally, we analysed the second species set, consisting of 102 samples (10 samples representing a new taxon, 10 of *S. arabica-hohenackeriana agg.*, 10 of *S. richteriana*, 10 of *S. capillata*, 10 of *S. orientalis*, 10 of *S. lessingiana*, 10 of *S. sareptana*, 10 of *S*. × *heptapotamica*, 10 of *S*. × *czerepanovii*, 10 of *S*. *korshinskyi*, 2 of *S*. × *pseudocapillata*) to differentiate our new taxon and the other hybrids along with parental species grow commonly in Kazakhstan. Data filtering steps for this dataset are the same as the previous one except for the threshold of call rates for each locus calculation (30%), and we excluded loci with reproducibility below 0.99. A total of 25,896 SNP loci were used to generate fastStructure analysis for K values from 1 to 10. The estimation of the best choice of K value was completed using the function in fastStructure software, and visualisation was done in Structure Plot v. 2.0^39^. Principal Component Analysis (PCA) for 91 samples plotted based on “gl.pcoa” function with Euclidean distance from the dartR package in RStudio. The results were visualized using these following packages: “plotly^40^”, “ggplot2^41^” and “ggthemes^42^”.

## Results

### Morphological analyses

**Fig. 1:**
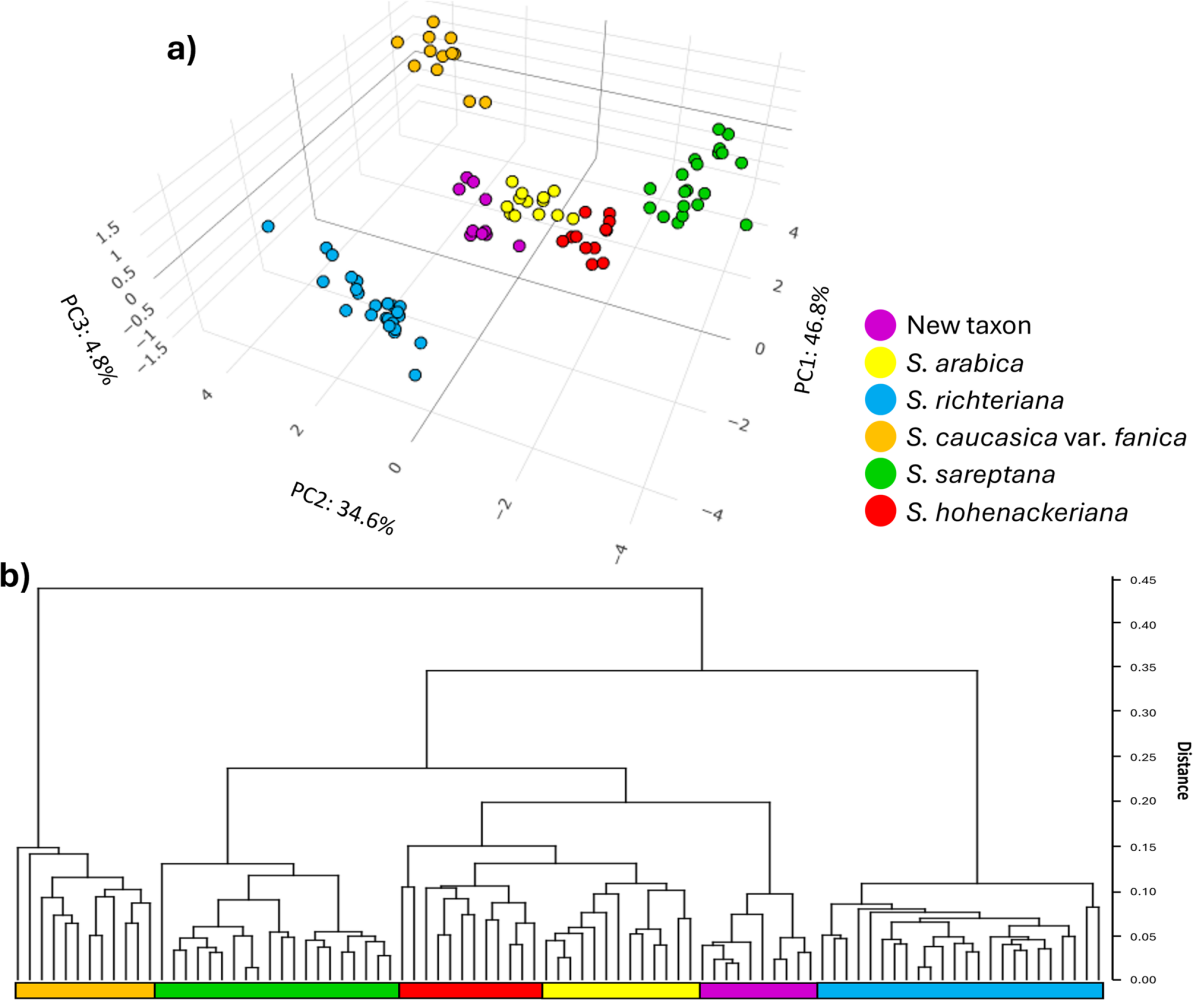
Morphological analyses of examined populations of feathergrasses: (**a**) PCA plot and (**b**) UPGMA dendrogram performed on 15 quantitative characters of 91 specimens of *Stipa.*

Principal Component Analysis (PCA) was conducted on five *Stipa* taxa based on 15 selected most informative quantitative morphological characters. The first three principal components accounted for 86.2% of the total morphological variance, with 46.8% explaining the first component, 34.6% for the second component and 4.8% for the third component. Ten characters were associated with the first axis and displayed positive factor loadings (> 0.6), whereas five characters were associated with the second axis and exhibited negative factor loadings (< -0.6) (Supplementary Table S2). We presented post-hoc comparisons for 15 morphological characters used in PCA, which revealed statistically significant differences between *Stipa* taxa with corresponding p-values < 0.05 (Supplementary Table S3). The PCA results demonstrate five distinctly distributed OTU groups, corresponding to the description of the following taxa: *S. arabica, S. hohenackeriana*, *S. richteriana*, *S. caucasica* var*. fanica*, *S. sareptana*, and the putative new taxon (Fig. 1a).

PCA plot based on three principal components showed the distribution of the OTUs of the putative new taxon intermediately between OTUs of *S. arabica* and *S. richteriana* (Fig. 1a). This distribution suggests a phenotypic combination between *S. arabica* and *S. richteriana*. The UPGMA dendrogram, based on 15 quantitative morphological characters, supports the species delimitation by exposing distinct clusters among *Stipa* taxa. The dendrogram displays distinct clades corresponding to the descriptions of analysed taxa. The putative new species is a sister to the *S. arabica-hohenackeriana* clade, which is most similar morphologically of all the examined species (Fig. 1b).

### Molecular analyses based on SNPs

**Fig. 2:**
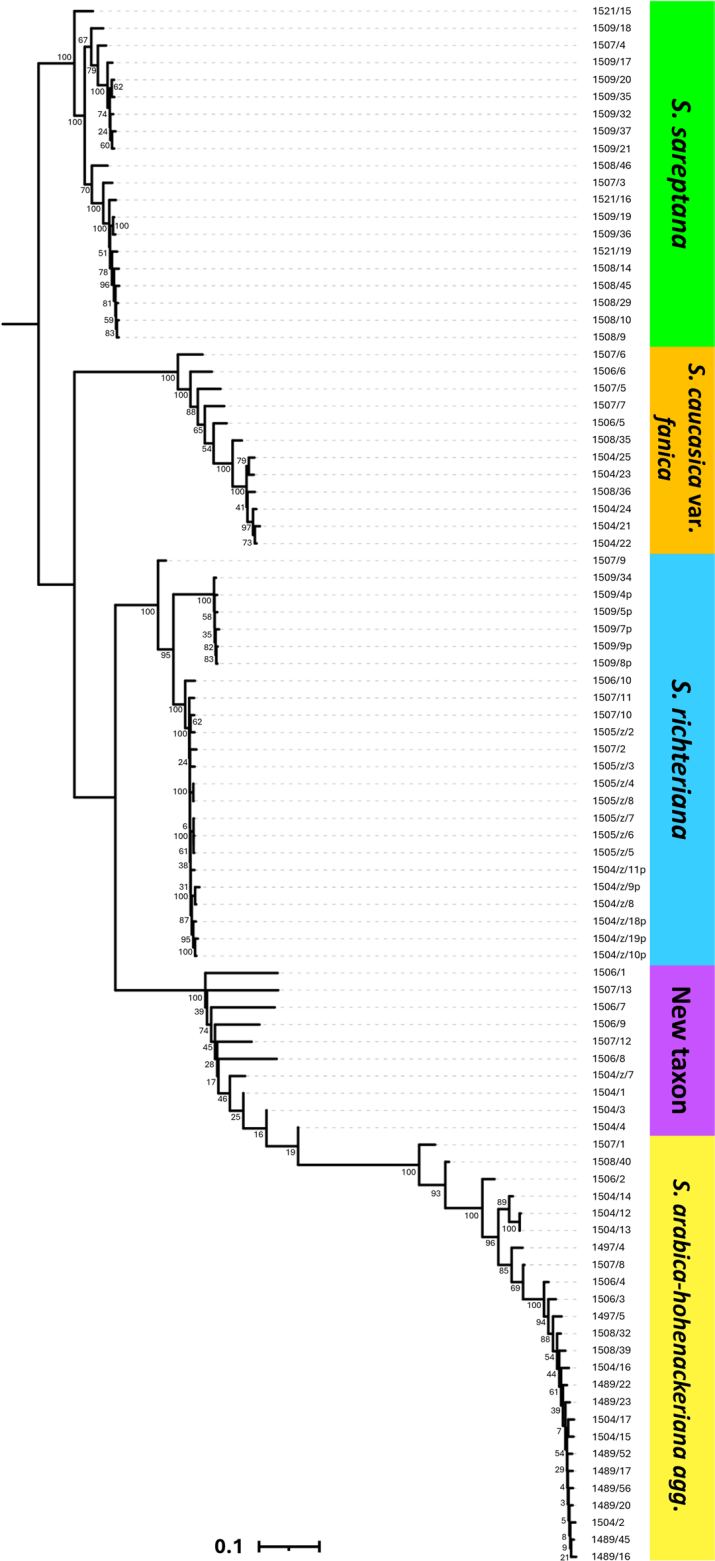
Neighbor-joining tree based on SNP markers of *Stipa* examined. A list of the samples is given in Supplementary Table S1.

The *Stipa* taxa showed clear clustering according to the neighbor-joining tree based on 3,444 SNP loci (Fig. 2). The analysis grouped *S. arabica* and *S. hohenackeriana* into the common clade, although most of *S. hohenackeriana* samples were grouped monophyletically in one of the subclades (except 1504/16 and 1504/17 which are closer to *S. arabica*). Despite both taxa differing quite well in the awn morphology (lower part of awn pilose in *S. arabica* vs. scabrous in *S. hohenackeriana*), molecular data do not segregate them into separate clades, thus both are treated as one entity to reduce the complexity of analysis and interpretation. Whereas the samples of the new taxon are scattered intermediately between *S. richteriana* and *S. arabica-hohenackeriana agg*.

**Fig. 3:**
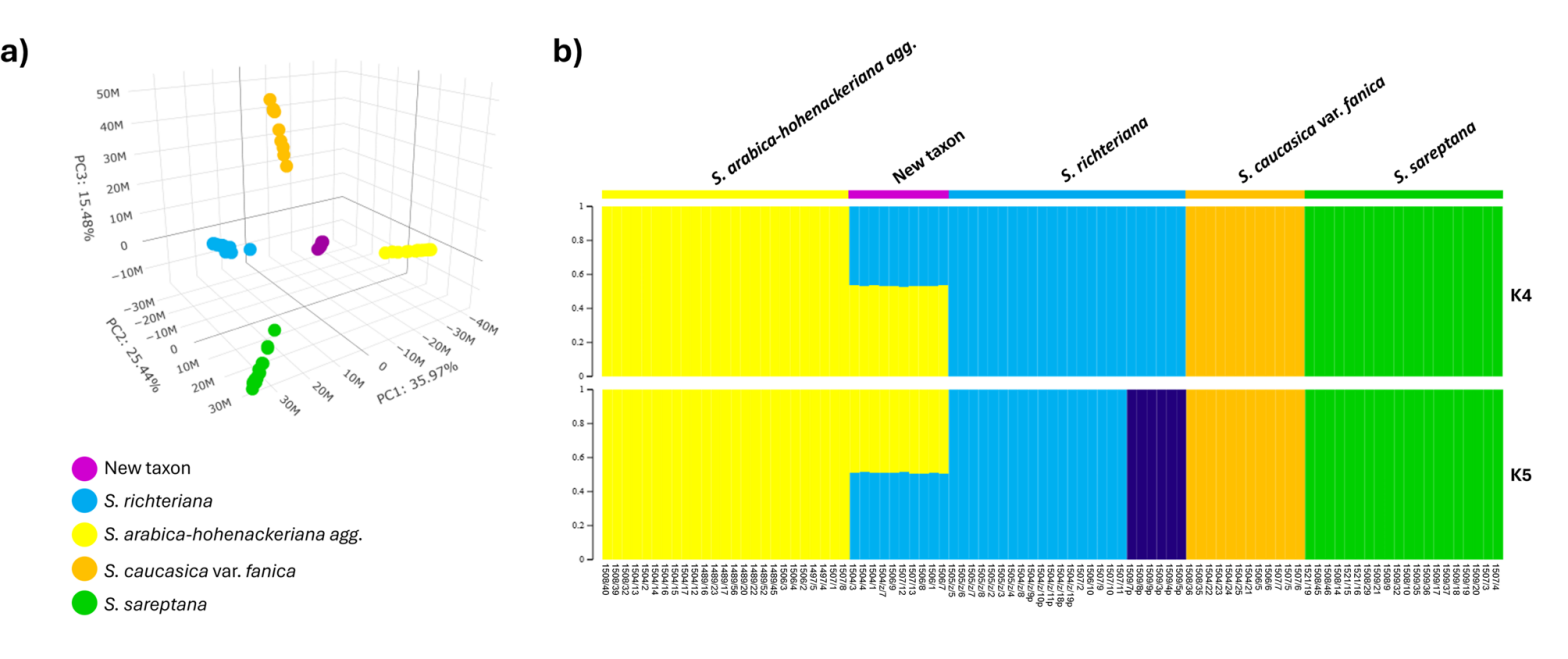
Genetic relationships and admixture of *Stipa* examined. (**a**) PCA and (**b**) fastStructure plot analysis based on 3,444 loci SNP markers of *S. arabica*-*hohenackeriana agg.*, *S. richteriana*, *S. caucasica* var. *fanica*, *S. sareptana*, and new taxon.

Principal Component Analysis (PCA) based on SNP markers revealed the genetic relationships among *S. arabica-hohenackeriana agg.*, *S. richteriana*, *S. caucasica* var. *fanica*, *S. sareptana* and the new taxon. The first three principal components described 76.89% of the genetic variation (PC1 35.97%, PC2 25.44%, and PC3 15.48%). PCA plot visualised the distribution of five taxa that were separated from each other, with the distribution of new taxon located in the central part of the plot (Fig. 3a).

Genetic admixture of *S. arabica-hohenackeriana agg.*, *S. richteriana*, *S. caucasica* var*. fanica*, *S. sareptana*, and the new taxon, involving a total of 91 samples indicated that K = 5 as the best clusters. All samples identified as new taxon displayed a unique admixture of approximately equal proportions of genetic contributions from *S. arabica-hohenackeriana agg.* and *S. richteriana*. This proportion suggests that all specimens are F1 hybrids, resulting from hybridisation between the two taxa of *S. arabica-hohenackeriana agg.* and *S. richteriana*. The fastStructure results support the neighbor-joining phylogenetic tree showing no distinct genetic differentiation between *S. arabica* and *S. hohenackeriana*, where in both taxa were clustered together suggesting a lack of genetic separation despite their morphological differences. Another interesting result of this analysis is the detection of two subpopulations of *S. richteriana* that are in line with the neighbor-joining tree, which was not visible when we accept the value of K = 4 (Fig. 3b). With these results, we believe that there are two genetic groups for *S. richteriana*, occurring in the steppe areas of Kazakhstan, however, they distribution seem to be genotype-specific; the dark blue samples represent more eastern genotype and the light blue genotype occur in more western part of the region.

### Hybrid identification

Molecular data have provided evidence of gene flow between *S. arabica-hohenackeriana agg.* and *S. richteriana*, classifying part of the examined species as an F1 hybrid. Having in mind that in Kazakhstan there were described a few more nothotaxa, of which one of the parental species is also *S. richteriana* we compared its most informative morphological characters with the characters of other taxa, such as: *S*. × *heptapotamica*, *S*. × *czerepanovii*, *S*. *korshinskyi*, *S*. × *pseudocapillata*. Here, we present key morphological characters that distinguish the new species, hereafter treated and named as *S.* × *kyzylordensis*, from other species of hybrid origin (Table 2).

**Table 2:** The comparison of the main morphological characters of *S.* × *kyzylordensis* with other species of hybrid origin.

Morphological character	*Stipa* × *kyzylordensis*	*Stipa* × *heptapotamica*	*Stipa* × *czerepanovii*	*Stipa* × *korshinskyi*	*Stipa* × *pseudocapillata*
Length of internal ligule of vegetative shoot (mm)	1.5–4.0(–6.0)	0.10–0.3	(0.5–)0.7–1.2(–1.4)	0.1–0.2	(0.5–)1–2
Length of hairs on adaxial surface of vegetative leaf (mm)	(0.2–)0.3–0.4	0.10–0.15(–0.20)	0.15–0.25(–0.30)	0.1–0.2	(0.1–)0.2–0.3
Awn length (cm)	(9.5–)10.0–11.5(–12.4)	8.5–14.0(–15.0)	(4.6–)5.0–7.5(–8.0)	(7.8–)8.5–12.0(–13.5)	16–20
Length of hairs on seta (mm)	(0.7 –)0.9–1.1	(0.8–)1.0–1.5	1.0–1.8(–2.0)	0.2–0.4	0.4–0.6(–0.7)
Length of hairs on column (mm)	0.5–0.6	0.1–0.2(–0.3)	(0.2–)0.3–0.5(–0.6)	0.1–0.2	0.1–0.2(– 0.3)
Floret (= anthecium) length (mm)	8.7–10.1	7.5–9.0	6.0–7.0(–7.5)	(7.4–)8.0–8.7	9–11
Callus length (mm)	1.1–1.2	(1.0–)1.3–1.6	0.9–1.3	1.3–1.7(–1.9)	2.0–2.3
Lower glume (mm)	17–23	15–20(–21)	12–16	12–16(–18)	22–26

**Fig. 4:**
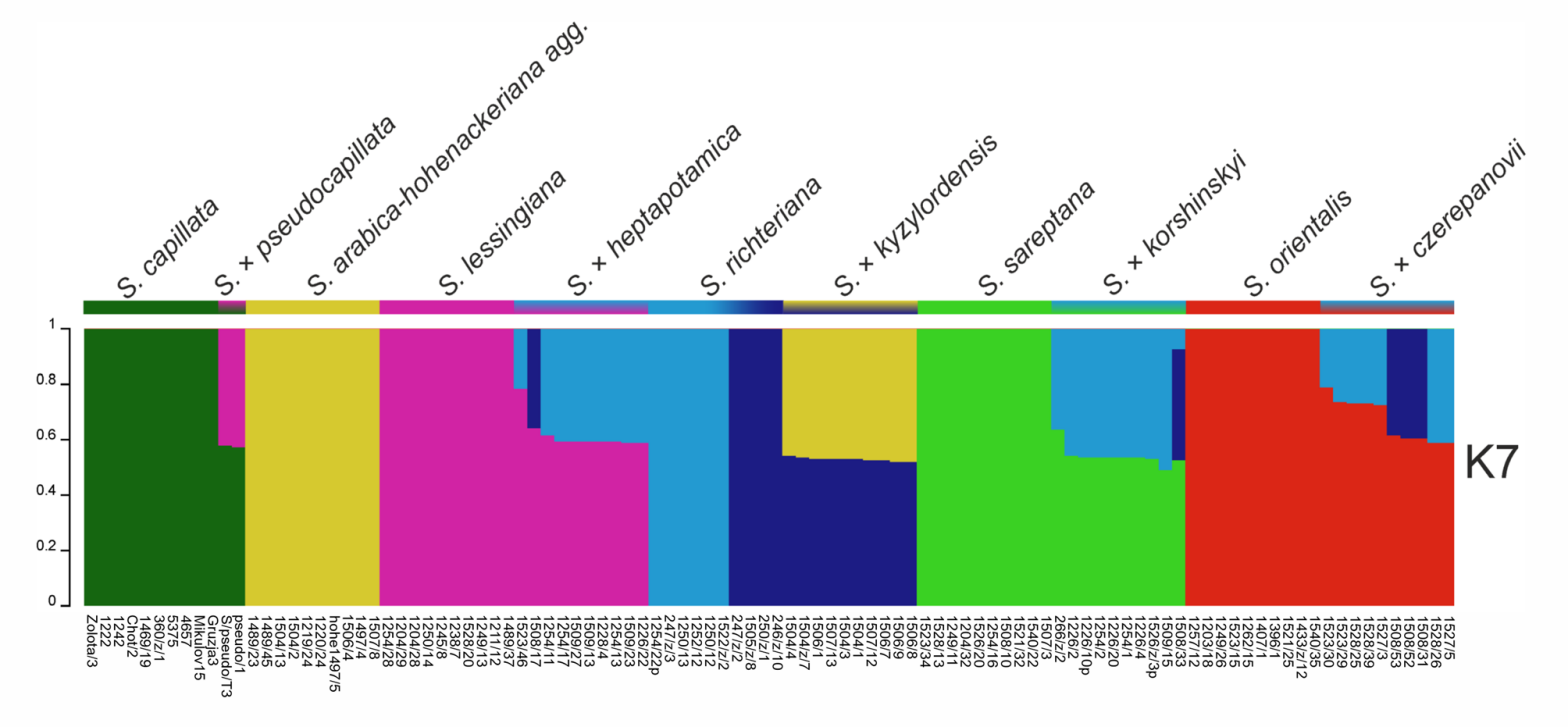
Genetic admixture of five *Stipa* hybrids along with parental species based on 25,896 SNP loci.

To be sure that the new taxon clearly differs from the above-mentioned taxa, we also conducted a genetic structure analysis based on 25,896 SNP loci. Besides hybrid origin taxa, we added the putative parental species, thus in the analysis we involved six species (*S. capillata*, *S. orientalis*, *S. arabica–hohenackeriana agg.*, *S. sareptana*, *S. lessingiana* and *S. richteriana*) and 42 samples representing taxa of hybrid origin (*S.* × *kyzylordensis, S*. × *heptapotamica*, *S*. × *czerepanovii*, *S*. *korshinskyi*, *S*. × *pseudocapillata*) with the best value of K = 7. Our findings also showed the existence of two genotypes of *S. richteriana*, similarly to the genetic structure results that we have mentioned before. It can be seen that both genotypes of *S. richteriana* (Fig. 4) were involved in the hybridisation processes with five analysed species (*S. orientalis*, *S. lessingiana*, *S. sareptana*, and *S. arabica*-*hohenackeriana* agg). The first, ‘western’ genotype, was found mainly around Balkhash Lake, while the second ‘eastern’, occurs in the northern foothills of the Tian-Shan Mountains. The eastern genotype of *S. richteriana* was identified as the parent in the majority of hybrid samples. Interestingly, one sample of *S*. × *heptapotamica* (hept1508/17) and three samples of *S*. × *czerepanovii* (× czerep1508/52, × czerep1508/53, and × czerep1508/31) showed that one of their parents was also the western *S. richteriana* genotype. In addition, one sample of *S*. *korshinskyi* (korsh1508/33) had an unbalanced genetic admixture, consisting of 53% of *S. sareptana*, 40% of the eastern *S. richteriana* genotype, and 7% of the western *S. richteriana* genotype, reflecting a more complicated scenario on the emergence of this hybrid. Meanwhile, only the western genotype of *S. richteriana* showed genetic admixture in *S.* × *kyzylordensis*, which tended to be almost equal in proportion with *S. arabica–hohenackeriana agg*. In addition, based on SNP markers, we confirmed that *S.* × *kyzylordensis* is different from other morphologically similar taxa of hybrid origin. Moreover, for the first time we evidenced the hybrid origin of *S*. × *pseudocapillata* (hybrid between *S*. *lessingiana* and *S*. *capillata*) as well as *S*. × *korshinskyi* (hybrid between *S. sareptana* and *S. richteriana*).

### Hybrid description

**Fig. 5:**
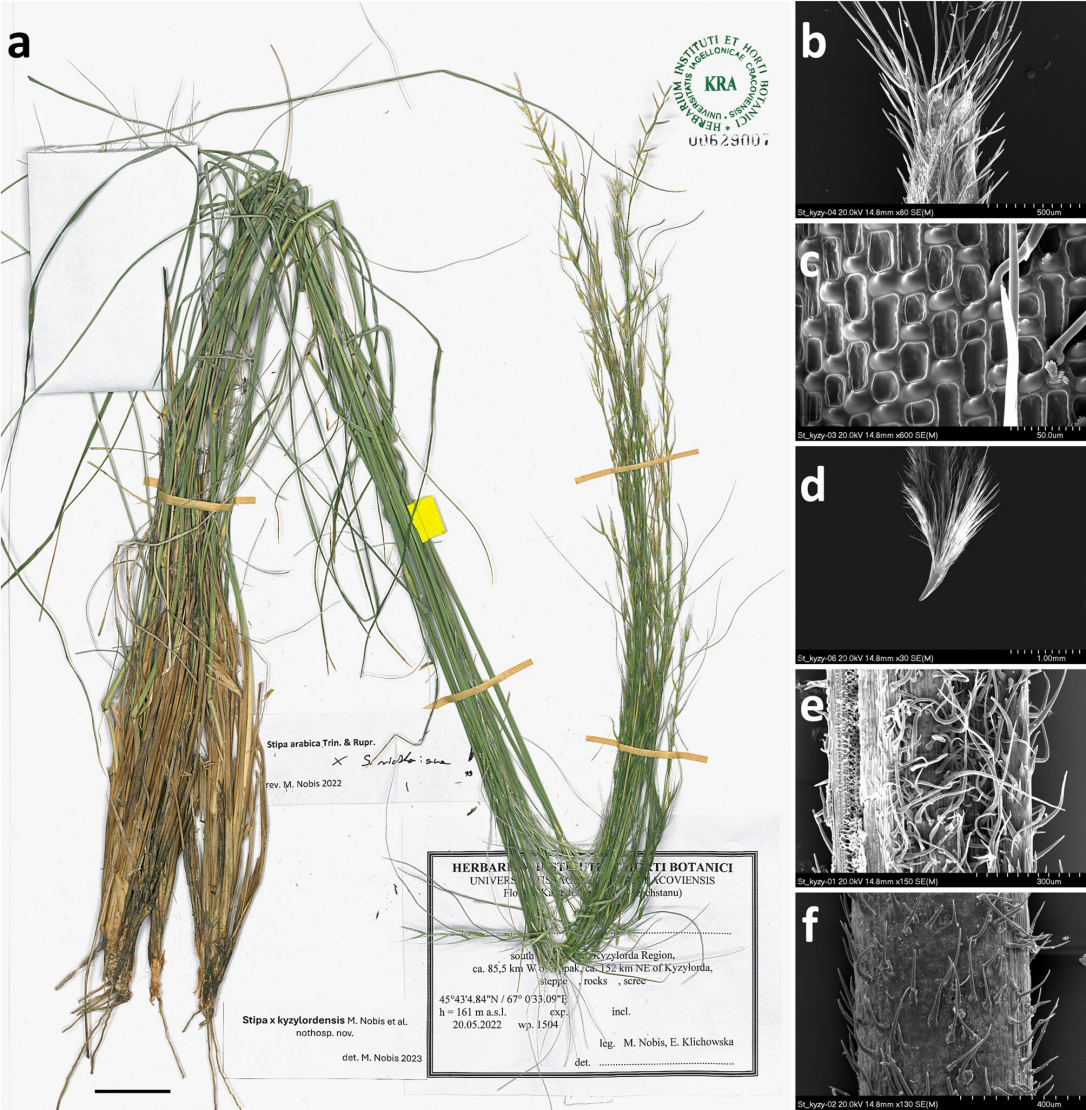
*S.* × *kyzylordensis M. Nobis, Klichowska, P. Sinaga.* (**a**) holotype, (**b**) top of the lemma, (**c**) patterns of the lemma micromorphology, (**d**) callus, (**e**) adaxial surface of the vegetative leaf, (f) abaxial surface of the vegetative leaf. Scale bars: (**a**) 3 cm, (**b**) 500 μm, (**c**) 50 μm, (**d**) 1 mm, (**e**) 300 μm, (**f**) 400 μm.

*Stipa* × *kzylordensis* M. Nobis, Klichowska & P. Sinaga, *nothosp. nov*. (*S. arabica–hohenackeriana agg.* × *S. richteriana* Kar. & Kir. subsp. *richteriana*). Type: South Kazakhstan, Kyzylorda region, ca. 85.5 km west of Appak, ca. 152 km northeast of Kyzylorda, semi dessert/steppe, 45˚43′ 4.84″ N/67˚0′ 33.09″ E, elev. 161 m.a.s.l., 20 May 2022, wp. 1504, *M. Nobis*, *E. Klichowska* s.n. (holotype KRA 00629007, isotype KRA 640344, KRA 00628991, KRA 640345, KRA 00629006, KRA 00629008; Fig. 5a).

#### Description

Plant perennial, tufted, with numerous culms and vegetative shoots; *culms* (71–)85–101 cm tall, 3-noded, mostly glabrous at upper nodes and densely pilose with down-directed hair at the lower nodes. *Leaves of vegetative shoots*: sheaths covered by hairs, ciliated at margins; *ligules* acute, inner ligules in tuft of vegetative shoot 1.5–4.0(–6.0) mm long, densely ciliated at apex, cilia 0.3–0.4(–0.6) mm long, outer ligules in tuft of vegetative shoot 0.5–2.0 mm long, densely ciliated at the apex, cilia 0.2–0.4 mm long; *blades* convolute, 0.7–1.1 mm in diameter, adaxial surface densely pilose with hairs (0.2–)0.3–0.4 mm long (Fig. 5e), abaxial surface pubescent with hairs up to 0.4 mm long (Fig. 5f). *Cauline leaves*: upper sheaths glabrous or slightly scabrous, lower sheaths densely pubescent with white edge; *ligules* obtuse, 0.7–1.5 mm long, densely ciliated at the apex, cilia 0.3–0.6 mm long, uppermost blades 43–160 mm long and ca. 0.7 mm in diameter. *Panicle* 31–36.5 cm long, branches erect, setulose, glumes subequal, lower glume 16–21 mm long, upper glume 17–23 mm long; lanceolate and scabrous with sparse cilia along the middle vein. *Florets (anthecia, lemma* + *callus)* 8.7–10.1 mm long and 0.7–0.8 mm wide, *lemma* covered by ascending hairs organized in 7 lines with scattered irregular prickles and short hairs between each line (Fig. 5c), hairs in the dorsal line 0.5–0.9 mm long and in the ventral line 0.4–0.6 mm long, the dorsal line of hairs terminates in 1–1.5 mm below the apex, while the ventral line terminates in 0.3–0.7 mm below the apex, at the top of lemma there are developed corolla of unequal hairs 0.8–1.1 mm long (Fig. 5b), *corolla* hairs started 0.5 mm below the apex developing from prickles–prickle–hairs–hairs; *palea* subequal to lemma in length, ovary with 3 stigmas. *Callus* 1.1–1.2 mm long, straight, densely pilose with ventral hairs (1.1–)1.4–1.6 mm long and dorsal hairs 0.8–1.2 mm long, foot of callus with peripheral ring ca. 0.7 mm long and 0.2 mm wide (Fig. 5d). *Awn* (9.5–)10.0–11.5(–12.4) cm long, bigeniculate; *lower segment of awn (lower column)* 21–25 mm long, 0.25–0.3 mm wide near basal, twisted, with hairs 0.5–0.6 mm long; *middle segment of awn (upper column)* 13–16 mm long covered by hairs 0.5–0.7 mm long; *upper part of awn (seta)* flexuous 58–66 mm long, hairs in the lower part of seta straight, (0.7 –)0.9–1.1 mm long gradually decreasing in length toward the apex.

Paratypes: South Kazakhstan, Kyzylorda/Ulytau region, semi dessert/steppe, ca. 94 km northwest of Appak, ca. 193 km northeast of Kyzylorda. 46˚2′2.80″N / 67˚2′6.04″E, elev. 217 m.a.s.l., 20 May 2022, wp. 1506, *M. Nobis, E. Klichowska* s.n. (KRA 00628876, KRA 00628875, KRA 00628866, KRA 00628877, KRA 640330, KRA 640346, KRA 640340, KRA 640341, KRA 640342).

#### Phenology

Flowering in May and June.

#### Distribution

Central–western Kazakhstan, Kyzylorda region.

#### Etymology

This species is named after Kyzylorda region in Central–western Kazakhstan.

## Discussion

Hybridisation is a widespread phenomenon that plays a crucial role in evolution of plants^43–47^. Hybridisation and introgression can give rise to new lineages, such as through allopolyploid hybrid speciation, where two parental species with different ploidy numbers hybridize^48,49^ or contribute to transferring adaptive alleles to aid adaptation^50^. Recently, with the advent of genomic-based studies, the evidence for the existence of natural hybrids and phylogenetic construction has become more comprehensive. The use of multiple markers such as SNPs^16,26,51,52^, chloroplast and nuclear genomes^43,53–57^ helps to reveal hybridisation patterns and identify their evolutionary consequences.

Distant hybridisation can promote new varieties, but this type of hybridisation is considered less vigorous than hybridisation of more closely related species due to differences in genotype^58^. Compatibility in distant hybridisation in plants can be influenced by chromosome number^59^, morphology, and growth habit^60^, which affect fertility. Incompatibility is also common in hybridisation especially between tetraploid species and their diploid relatives^61^. Limitations of interspecific hybridisation may be due to imbalanced parental genome resulting in endosperm failure^62^. In peony, for example, the mechanism of compatibility in intersectional hybridisation is supposed to be a rebalance between divergent parents triggered by increased gamete ploidy of tree peony^63^. Another example is *Rhododendron*, although there are obvious pre–fertilization and post–fertilization barriers in the hybridisation of *Rhododendron* × *hybridum* hort. and *R*. *decorum*, which are distant hybridizers between subgenera, pre-fertilisation barriers can be overcome by early pollination and delayed pollination^58^. Similar situations can be observed in grasses such as *Elymus*, *Calamagrostis*, *Puccinellia* or *Stipa,* where the cases of hybridisation happened in both closely and distant related species^16,23,24,26,64–66^.

In *Stipa*, for instance, genetically closely related species *S. richteriana* and *S. lessingiana,* previously described as belonging to sections *Leiostipa* and *Barbatae* respectively, hybridised to form *Stipa* × *heptapotamica*^23^. Another example can be *S*. × *lazkovii*, which is a hybrid of *S. krylovii* and *S*. *bungeana*, two genetically distant species^16^ or *S.* × *smelanskyi* which has been identified as a hybrid between *S. richteriana* and *S. drobovii*, which represent sections *Leiostipa* and *Smirnovia*, respectively^67^*.* All the aforementioned hybrids were evidenced based on the integrative taxonomy approach combining morphological characters and wide–genome sequencing datasets. Here we documented an evidence of another new hybrid, *S*. × *kyzylordensis* (*S. arabica–hohenackeriana agg*. × *S. richteriana*). Of the parental taxa analyzed, *S. arabica* and *S. hohenackeriana* are classified under section *Barbatae*, which is characterized by ovaries with three or four unequal or equal stigmas on the ovary^68^ and long lanceolate ligules, and *S. richteriana,* which is classified in the section *Leiostipa* by having scabrous or shortly pilose awns and ovary with two stigmas. All section assignments follow the classification of Tzvelev^69^ based on *Stipa* that occurred in the former USSR.

Nowadays, molecular markers are crucial for clarifying the boundaries of species and uncovering genetic diversity masked by phenotypic plasticity, illuminating unsolved taxonomic issues. The samples of *S*. × *kyzylordensis* are F1 hybrid based on the 50:50 mixing ratio, which is a common pattern in hybrid zones. These results represent the first event of hybridisation of these two species (*S. arabica–hohenackeriana agg*. × *S. richteriana*). Additionally, similarly to the results of Nobis et al.^23^, our phylogenetic analysis and genetic admixture showed the presence of two genotypes of *S. richteriana*, reflecting genetic differentiation and separating the samples into two groups with slightly different geographical distributions. The first occurs in Kyzylorda and Ulytau regions and the second occurs in Karaganda region. Further studies are needed to understand these differentiation patterns in genetic structure and morphological patterns of *S. richteriana* s. lato and this is a challenge of our next studies (Klichowska et al. in prep.).

*Stipa* × *kyzylordensis* is morphologically and genetically well–differentiated from the other taxa that originated from hybridisation of *Stipa* species that are common in the study area (Table 2, Fig. 4). According to Nobis et al.^21^, *S*. × *pseudocapillata* is most likely the hybrid origin of *S. lessingiana* and *S. sareptana,* as in the locality where *S. x pseudocapillata* was collected, the two species were also present^21^. Moreover, they flower at the same time, which is in May and June. However, based on SNP markers, we successfully confirmed that *S.* × *pseudocapillata* is a hybrid between *S. lessingiana* and *S. capillata* and confirmed the hypothesis of Tzvelev^69^. This case supports that occasionally *S. capillata* could flower earlier, and then the gene flow (pollen exchange) between the two mentioned above taxa can occur. The two samples showed genetic admixture around 43% for *S. lessingiana* and 57% for *S. capillata*. Additionally, our results also supported hybrid origin of *S*. *korshinskyi* (*S. richteriana* × *S. sareptana*), which previously was also suggested by Nobis et al.^21^ however here we evidenced that both genotypes of *S. richteriana* were involved with this hybridisation processes. The genetic structure (Fig. 4) also successfully identified the presence of hybridisation between *S. lessingiana* and *S. richteriana*. This finding is in line with research conducted by Nobis et al.^23^, which was based on morphological analysis and molecular data using the inter simple sequence repeat (ISSR) and intergenic spacer (IGS) methods that have proven that *S*. × *heptapotamica* is a hybrid origin instead of a “good” species as previously described. It is also worth to mentioning, that the existence of two genetic groups within *S. richteriana*, both involved in the hybridization processes with *S. lessingiana*, has also been detected by Nobis et al.^23^.

We also found introgressive hybridisation events between first-generation hybrids and the eastern genotype of *S. richteriana*. For instance, in *S*. × *heptapotamica*, one introgressant (hept1523/46) was found displaying an unbalanced genetic admixture, which was about 78% of *S*. *lessingiana* and 22% of *S*. *richteriana*. A similar result of *S*. × *heptapotamica* was also reported in several samples as introgressive hybrids between *S*. *lessingiana* and the first generation hybrid in Nobis et al.^23^. Furthermore, several samples suspected of introgression were identified in five *S*. × *czerepanovii* samples (× czerep1528/39, × czerep1528/25, × czerep1523/30, × czerep1523/29, and × czerep1527/3), which > 73% genetic admixture were dominated by *S*. *orientalis* and in one *S*. *korshinskyi* sample (korsh266/z/2) that showed a genetic admixture of 63% from *S*. *sareptana*. Recently, introgression in *Stipa* was also reported in *S*. × *ochyrae*^26^ as the result of a backcross between a first-generation hybrid and *S*. *magnifica*. Detection of introgression is very important in plant hybridisation studies, not only to shed the light on the hybrid formation but also to reveal its consequences that can introduce new alleles and contribute to the adaptation to the new environment as well as to climate changes^70,71^.

This study provides a compelling example of how *S. arabica* and *S. hohenackeriana*, despite exhibiting clear morphological differences—particularly in the lower part of awn, which is pilose in *S. arabica* and scabrous in *S. hohenackeriana*—are classified as a single genetic entity based on SNP markers indicating no obvious distinction between the two taxa (Fig. 3). Notably, a comparison of nucleotide differences based on plastid barcode reveals only 17 bases separating *S. arabica* from *S. hohenackeriana*^72^, indicating very low genetic diversity within their plastome and this is exemplified by having almost complete lack of nucleotide differences (0.012%) in the analysis based on cpDNA^21,72,73^. Even though these two species are genetically close to each other, the notable morphological differences between them emphasize the phenomena of phenotypic divergence that can arise when there is no clear genetic distinction. In order to reconcile the status of *S. arabica* and *S. hohenackeriana*, which is an intriguing phenomenon, further research using new methods or their combinations are needed.

## Electronic supplementary material

Below is the link to the electronic supplementary material.

Supplementary Material 1

Supplementary Material 2

Supplementary Material 3

## Data Availability

The datasets generated and/or analysed during the current study (SNP datasets derived from the DArTseq pipeline in the genlight format) are available in the Figshare repository, [10.6084/m9.figshare.28532627.v2].
