# Supplementary material for: Integrated genomics and morphological approach reveals interspecific gene flow cases and decodes the origin of selected feathergrasses (Poaceae, Stipa)
Source: Sci Rep. 2025 Oct 1;15:34144. doi: 10.1038/s41598-025-08934-y (PMC12489067; doi:10.1038/s41598-025-08934-y)
Supplement: Supplementary file 2 — Supplementary Material 2 [file 41598_2025_8934_MOESM2_ESM.docx]

**Table S2**. Results of the Principal Component Analysis (PCA) of 6 *Stipa* taxa (*S. arabica*, *S. hohenackeriana,* *S. richteriana*, *S. caucasica* var. *fanica*, *S. sareptana*, and new taxon). PCA was based on 15 morphological quantitative characters (with factor loading > 0.65), Kruskal-Wallis test with H and ANOVA F (indicated with ^*^) value all with p value <0.05.

| **Character** | **PC1** | **PC2** | **PC3** | **F/H value** |
| --- | --- | --- | --- | --- |
| Lower column length | **-0,626** | 0,592 | -0,443 | 77.82 |
| Length of hairs on column | -0,469 | **-0,796** | -0,207 | 85.96 |
| Seta (upper segment of the awn) length | **-0,766** | 0,594 | -0,023 | 138.6^*^ |
| Length of hairs on seta | **-0,691** | -0,680 | 0,136 | 84.96 |
| Awn length | -0,681 | **0,687** | -0,105 | 161.4^*^ |
| Ratio of seta length to column length | **-0,882** | -0,104 | 0,222 | 73.87 |
| Floret (=anthecium) length | **-0,964** | 0,031 | 0,089 | 79.61 |
| Length of ventral hairs on lemma | -0,005 | **-0,751** | -0,107 | 35.97 |
| Corolla hairs length | -0,001 | **-0,899** | -0,258 | 78.29 |
| Callus length | **-0,780** | 0,382 | -0,404 | 84.74 |
| Callus width | -0,175 | **-0,825** | -0,239 | 51.4 |
| Callus foot ring length | -0,167 | **-0,794** | 0,022 | 53.99 |
| Upper glume length at the end of panicle | **-0,936** | -0,228 | 0,103 | 80.75 |
| Lower glume length at the end of panicle | **-0,937** | -0,233 | 0,115 | 81.76 |
| Length of hairs on adaxial surface of vegetative leaf | **-0,865** | 0,032 | 0,273 | 68.82 |
