# Supplementary material for: Integrated genomics and morphological approach reveals interspecific gene flow cases and decodes the origin of selected feathergrasses (Poaceae, Stipa)
Source: Sci Rep. 2025 Oct 1;15:34144. doi: 10.1038/s41598-025-08934-y (PMC12489067; doi:10.1038/s41598-025-08934-y)
Supplement: Supplementary file 3 — Supplementary Material 3 [file 41598_2025_8934_MOESM3_ESM.docx]

**Table S3.** Post-hoc p-values of 15 morphological characters. Post-hoc analysis conducted using Tukey’s pairwise (indicated as *) and Dunn’s post hoc with Bonferroni p values. Significant comparisons are in green. Abbreviations for each character as listed in Table 1.

| **LCL** | ***S. fanica*** | ***S. richteriana*** | **New taxon** | ***S. arabica*** | ***S. hohenackeriana*** | ***S. sareptana*** |
| --- | --- | --- | --- | --- | --- | --- |
| ***S. fanica*** |  | 2,11E-02 | 1 | 1 | 1 | 0.2077 |
| ***S. richteriana*** | 2,11E-02 |  | 0.00746 | 0.07165 | 0.0006452 | 1.18E-16 |
| **New taxon** | 1 | 0.00746 |  | 1 | 1 | 0.01257 |
| ***S. arabica*** | 1 | 0.07165 | 1 |  | 1 | 6,90E-02 |
| ***S. hohenackeriana*** | 1 | 0.0006452 | 1 | 1 |  | 0.02277 |
| ***S. sareptana*** | 0.2077 | 1.18E-16 | 0.01257 | 6,90E-02 | 0.02277 |  |
|  | | | | | | |
| **CH** | ***S. fanica*** | ***S. richteriana*** | **New taxon** | ***S. arabica*** | ***S. hohenackeriana*** | ***S. sareptana*** |
| ***S. fanica*** |  | 0.0001606 | 0.6825 | 1 | 2,66E-09 | 7,73E-07 |
| ***S. richteriana*** | 0.0001606 |  | 0.9429 | 0.04191 | 0.0006147 | 0.08188 |
| **New taxon** | 0.6825 | 0.9429 |  | 1 | 7,66E-03 | 0.001034 |
| ***S. arabica*** | 1 | 0.04191 | 1 |  | 8,78E-06 | 2,27E-03 |
| ***S. hohenackeriana*** | 2,66E-09 | 0.0006147 | 7,66E-03 | 8,78E-06 |  | 1 |
| ***S. sareptana*** | 7,73E-07 | 0.08188 | 0.001034 | 2,27E-03 | 1 |  |
|  | | | | | | |
| **SL^*^** | ***S. fanica*** | ***S. richteriana*** | **New taxon** | ***S. arabica*** | ***S. hohenackeriana*** | ***S. sareptana*** |
| ***S. fanica*** |  | 0.0001237 | 0.02785 | 0.2958 | 0.0009154 | 0.0001237 |
| ***S. richteriana*** | 0.0001237 |  | 0.0001237 | 0.0001237 | 0.0001237 | 0.0001237 |
| **New taxon** | 0.02785 | 0.0001237 |  | 0.0001402 | 0.0001237 | 0.0001237 |
| ***S. arabica*** | 0.2958 | 0.0001237 | 0.0001402 |  | 0.2437 | 0.0001238 |
| ***S. hohenackeriana*** | 0.0009154 | 0.0001237 | 0.0001237 | 0.2437 |  | 0.006513 |
| ***S. sareptana*** | 0.0001237 | 0.0001237 | 0.0001237 | 0.0001238 | 0.006513 |  |
|  | | | | | | |
| **SH** | ***S. fanica*** | ***S. richteriana*** | **New taxon** | ***S. arabica*** | ***S. hohenackeriana*** | ***S. sareptana*** |
| ***S. fanica*** |  | 2,79E-04 | 0.0138 | 1 | 0.6251 | 9,84E-11 |
| ***S. richteriana*** | 2,79E-04 |  | 1 | 0.0006637 | 0.01597 | 0.06984 |
| **New taxon** | 0.0138 | 1 |  | 0.7001 | 1 | 0.003454 |
| ***S. arabica*** | 1 | 0.0006637 | 0.7001 |  | 1 | 3,17E-06 |
| ***S. hohenackeriana*** | 0.6251 | 0.01597 | 1 | 1 |  | 5,22E-04 |
| ***S. sareptana*** | 9,84E-11 | 0.06984 | 0.003454 | 0 | 0 |  |
|  | | | | | | |
| **Awn^*^** | ***S. fanica*** | ***S. richteriana*** | **New taxon** | ***S. arabica*** | ***S. hohenackeriana*** | ***S. sareptana*** |
| ***S. fanica*** |  | 0.0001237 | 1 | 0.00311 | 0.0001243 | 0.0001237 |
| ***S. richteriana*** | 0.0001237 |  | 0.0001237 | 0.0001237 | 0.0001237 | 0.0001237 |
| **New taxon** | 1 | 0.0001237 |  | 0.003628 | 0.0001249 | 0.0001237 |
| ***S. arabica*** | 0.00311 | 0.0001237 | 0.003628 |  | 0.2641 | 0.0001237 |
| ***S. hohenackeriana*** | 0.0001243 | 0.0001237 | 0.0001249 | 0.2641 |  | 0.0001241 |
| ***S. sareptana*** | 0.0001237 | 0.0001237 | 0.0001237 | 0.0001237 | 0.0001241 |  |
|  | | | | | | |
| **RatioSC** | ***S. fanica*** | ***S. richteriana*** | **New taxon** | ***S. arabica*** | ***S. hohenackeriana*** | ***S. sareptana*** |
| ***S. fanica*** |  | 1,82E-09 | 3,22E-02 | 0.6248 | 1 | 0.008652 |
| ***S. richteriana*** | 1,82E-09 |  | 1 | 2,34E-03 | 9,57E-04 | 9,82E-02 |
| **New taxon** | 0 | 1 |  | 0.05855 | 0.03089 | 0.692 |
| ***S. arabica*** | 0.6248 | 2,34E-03 | 0.05855 |  | 1 | 1,00E+00 |
| ***S. hohenackeriana*** | 1 | 9,57E-04 | 0.03089 | 1 |  | 1,00E+00 |
| ***S. sareptana*** | 0.008652 | 9,82E-02 | 0.692 | 1 | 1 |  |
|  | | | | | | |
| **AL** | ***S. fanica*** | ***S. richteriana*** | **New taxon** | ***S. arabica*** | ***S. hohenackeriana*** | ***S. sareptana*** |
| ***S. fanica*** |  | 1,75E-10 | 1,71E-02 | 0.2393 | 1 | 0.004879 |
| ***S. richteriana*** | 1,75E-10 |  | 1 | 4.48E-06 | 5,66E-05 | 4,30E-02 |
| **New taxon** | 0 | 1 |  | 0.1174 | 0.01178 | 0.6977 |
| ***S. arabica*** | 0.2393 | 4.48E-06 | 0.1174 |  | 1 | 1,00E+00 |
| ***S. hohenackeriana*** | 1 | 5,66E-05 | 0.01178 | 1 |  | 1,00E+00 |
| ***S. sareptana*** | 0.004879 | 4,30E-02 | 0.6977 | 1 | 1 |  |
|  | | | | | | |
| **LHV** | ***S. fanica*** | ***S. richteriana*** | **New taxon** | ***S. arabica*** | ***S. hohenackeriana*** | ***S. sareptana*** |
| ***S. fanica*** |  | 0.564 | 0.1951 | 0.06862 | 0.00929 | 9,74E-05 |
| ***S. richteriana*** | 0.564 |  | 1 | 1,00E+00 | 0.9153 | 7,27E-02 |
| **New taxon** | 0.1951 | 1 |  | 1 | 1 | 0.09617 |
| ***S. arabica*** | 0.06862 | 1,00E+00 | 1 |  | 1 | 0.08616 |
| ***S. hohenackeriana*** | 0.00929 | 0.9153 | 1 | 1 |  | 0.7215 |
| ***S. sareptana*** | 9,74E-05 | 7,27E-02 | 0.09617 | 0.08616 | 0.7215 |  |
|  | | | | | | |
| **HC** | ***S. fanica*** | ***S. richteriana*** | **New taxon** | ***S. arabica*** | ***S. hohenackeriana*** | ***S. sareptana*** |
| ***S. fanica*** |  | 0.1596 | 1,00E+00 | 0.000144 | 7,01E-04 | 1,76E-09 |
| ***S. richteriana*** | 0.1596 |  | 1 | 0.1747 | 0.002609 | 3,65E-05 |
| **New taxon** | 1 | 1 |  | 0.1341 | 0.004101 | 2,16E-03 |
| ***S. arabica*** | 0.000144 | 0.1747 | 0.1341 |  | 1 | 0.1274 |
| ***S. hohenackeriana*** | 0 | 0.002609 | 0.004101 | 1 |  | 1,00E+00 |
| ***S. sareptana*** | 1,76E-09 | 3,65E-05 | 2,16E-03 | 0.1274 | 1 |  |
|  | | | | | | |
| **CalL** | ***S. fanica*** | ***S. richteriana*** | **New taxon** | ***S. arabica*** | ***S. hohenackeriana*** | ***S. sareptana*** |
| ***S. fanica*** |  | 1,09E-04 | 0.02819 | 0.2932 | 1,00E+00 | 1,00E+00 |
| ***S. richteriana*** | 1,09E-04 |  | 0.866 | 0.0189 | 0.0004781 | 1,24E-13 |
| **New taxon** | 0.02819 | 0.866 |  | 1 | 1 | 1,63E-02 |
| ***S. arabica*** | 0.2932 | 0.0189 | 1 |  | 1 | 0.0004225 |
| ***S. hohenackeriana*** | 1 | 0.0004781 | 1 | 1 |  | 0.02905 |
| ***S. sareptana*** | 1,00E+00 | 1,24E-13 | 1,63E-02 | 0.0004225 | 0.02905 |  |
|  | | | | | | |
| **CalW** | ***S. fanica*** | ***S. richteriana*** | **New taxon** | ***S. arabica*** | ***S. hohenackeriana*** | ***S. sareptana*** |
| ***S. fanica*** |  | 0.07571 | 0.001057 | 0.0425 | 2,62E-04 | 7,67E-07 |
| ***S. richteriana*** | 0.07571 |  | 0.8859 | 1,00E+00 | 0.003196 | 5,07E-02 |
| **New taxon** | 0.001057 | 0.8859 |  | 1 | 1 | 1,00E+00 |
| ***S. arabica*** | 0.0425 | 1,00E+00 | 1 |  | 0.08627 | 0.011 |
| ***S. hohenackeriana*** | 0 | 0.003196 | 1 | 0.08627 |  | 1,00E+00 |
| ***S. sareptana*** | 7,67E-07 | 5,07E-02 | 1,00E+00 | 0.011 | 1 |  |
|  | | | | | | |
| **CRL** | ***S. fanica*** | ***S. richteriana*** | **New taxon** | ***S. arabica*** | ***S. hohenackeriana*** | ***S. sareptana*** |
| ***S. fanica*** |  | 0.0007856 | 0.4226 | 0.03875 | 0.005297 | 5,06E-09 |
| ***S. richteriana*** | 0.0007856 |  | 1 | 1,00E+00 | 1,00E+00 | 0.0007518 |
| **New taxon** | 0.4226 | 1 |  | 1 | 1 | 0.0001375 |
| ***S. arabica*** | 0.03875 | 1,00E+00 | 1 |  | 1 | 0.0006929 |
| ***S. hohenackeriana*** | 0.005297 | 1,00E+00 | 1 | 1 |  | 0.01535 |
| ***S. sareptana*** | 5,06E-09 | 0.0007518 | 0.0001375 | 0.0006929 | 0.01535 |  |
|  | | | | | | |
| **UG** | ***S. fanica*** | ***S. richteriana*** | **New taxon** | ***S. arabica*** | ***S. hohenackeriana*** | ***S. sareptana*** |
| ***S. fanica*** |  | 9,17E-11 | 2.11E-05 | 1 | 0.5162 | 0.001202 |
| ***S. richteriana*** | 9,17E-11 |  | 0.9855 | 4,29E-05 | 1,26E-03 | 0.000201 |
| **New taxon** | 2.11E-05 | 0.9855 |  | 0.01952 | 0.07495 | 1 |
| ***S. arabica*** | 1 | 4,29E-05 | 0.01952 |  | 1 | 0.6183 |
| ***S. hohenackeriana*** | 0.5162 | 1,26E-03 | 0.07495 | 1 |  | 1 |
| ***S. sareptana*** | 0.001202 | 0.000201 | 1 | 0.6183 | 1 |  |
|  | | | | | | |
| **LG** | ***S. fanica*** | ***S. richteriana*** | **New taxon** | ***S. arabica*** | ***S. hohenackeriana*** | ***S. sareptana*** |
| ***S. fanica*** |  | 8,56E-11 | 2.08E-05 | 1 | 0.7162 | 0.0007304 |
| ***S. richteriana*** | 8,56E-11 |  | 0.9745 | 4,08E-05 | 4,96E-04 | 0.0003636 |
| **New taxon** | 2.08E-05 | 0.9745 |  | 0.01936 | 0.04932 | 1 |
| ***S. arabica*** | 1 | 4,08E-05 | 0.01936 |  | 1 | 0.46 |
| ***S. hohenackeriana*** | 0.7162 | 4,96E-04 | 0.04932 | 1 |  | 0.9678 |
| ***S. sareptana*** | 0.0007304 | 0.0003636 | 1 | 0.46 | 0.9678 |  |
|  | | | | | | |
| **LAH** | ***S. fanica*** | ***S. richteriana*** | **New taxon** | ***S. arabica*** | ***S. hohenackeriana*** | ***S. sareptana*** |
| ***S. fanica*** |  | 1,96E-06 | 0.03518 | 1 | 1 | 0.08394 |
| ***S. richteriana*** | 1,96E-06 |  | 0.1501 | 1,12E-05 | 1,13E-05 | 0.0004705 |
| **New taxon** | 0.03518 | 0.1501 |  | 0.09348 | 0.07243 | 1 |
| ***S. arabica*** | 1 | 1,12E-05 | 0.09348 |  | 1 | 0.2378 |
| ***S. hohenackeriana*** | 1 | 1,13E-05 | 0.07243 | 1 |  | 0.1824 |
| ***S. sareptana*** | 0.08394 | 0.0004705 | 1 | 0.2378 | 0.1824 |  |
|  | | | | | | |
